# Ten Years of Tau-Targeted Immunotherapy: The Path Walked and the Roads Ahead

**DOI:** 10.3389/fnins.2018.00798

**Published:** 2018-11-02

**Authors:** Petr Novak, Eva Kontsekova, Norbert Zilka, Michal Novak

**Affiliations:** ^1^Institute of Neuroimmunology, Slovak Academy of Sciences, Bratislava, Slovakia; ^2^AXON Neuroscience CRM Services SE, Bratislava, Slovakia; ^3^AXON Neuroscience R&D Services SE, Bratislava, Slovakia; ^4^AXON Neuroscience SE, Larnaca, Cyprus

**Keywords:** tau, immunotherapy, neurofibrillary degeneration, clinical trials, animal models

## Abstract

Neurofibrillary pathology comprised of pathological tau protein is closely tied to a range of neurodegenerative disorders, the most common of which is Alzheimer’s disease. While they are individually rarer, a range of other disorders, the tauopathies (including Pick’s disease, progressive supranuclear palsy, corticobasal degeneration, primary progressive aphasia, and ∼50% of behavioral variant frontotemporal dementia cases) display pronounced underlying tau pathology. In all cases, the distribution and amount of tau pathology closely correlates with the severity and phenotype of cognitive impairment, and with the pattern and degree of brain atrophy. Successfully counteracting tau pathology is likely to halt or slow the progression of these debilitating disorders. This makes tau a target of prime importance, yet an elusive one. The diversity of the tau proteome and post-translational modifications, as well as pathophysiology of tau are reviewed. Beginning 2013, a range of tau-targeted immunotherapies have entered clinical development; these therapies, and their common themes and differences are reviewed. The manuscript provides an extensive discussion on epitope selection for immunotherapies against tau pathology, on immunological mechanisms involved in their action, and challenges such as immune senescence, vaccine design, or evolution of epitopes. Furthermore, we provide methodological recommendations for the characterization of active vaccines and antibodies, animal models, and the target itself – the diseased tau proteome.

## Tau Pathology in a Nutshell

In his seminal discovery, Alois Alzheimer has described two prominent changes in the brain of his patient – senile plaques composed of amyloid-β, and neurofibrillary pathology composed of protein tau (Alzheimer, 1907; Braak et al., 1994). The former received massive attention due to the simple fact that rare monogenic mutations in the amyloid precursor protein, or in the presenilins involved in its processing cause a neurodegenerative phenotype that is indistinguishable from ‘sporadic’ AD that makes up >95% of AD cases (Blennow et al., 2006). The discovery that tau mutations are able to cause neurodegenerative disease on their own (Poorkaj et al., 1998) was vital to highlight the clinical relevance of tau pathology. Recent large-scale studies have revealed the close correlation between tau lesions and progression of neurodegenerative disease (Nelson et al., 2012; Murray et al., 2015). The disease phenotype reflects the distribution and severity of tau pathology in multiple tauopathies, including primary progressive aphasia, progressive supranuclear palsy, and corticobasal degeneration (Josephs et al., 2006). Analogously, tau PET imaging studies demonstrated that tau pathology is related in a region-specific manner to cognitive impairment in Alzheimer’s disease subjects (Bejanin et al., 2017). Interestingly, tau pathology in the medial temporal lobe is associated with episodic memory performance and atrophy also in cognitively normal adults, independent of Aβ (Maass et al., 2018). Tau pathology is a condition sine qua non for Alzheimer’s-type dementia, present in some form from the inception of the neurodegenerative process to its grim conclusion (Braak and Braak, 1991; Braak and Del Tredici, 2011); pure amyloidosis is asymptomatic (Murray et al., 2015). It is not far-fetched to expect that successfully counteracting the progression of tau pathology would delay the progression of AD as a whole, or halt it in its tracks if an efficacious intervention is applied early enough.

The main challenge in the study of tau pathology is the immense diversity of the pool of physiological and pathological tau molecules. Alternative splicing gives rise to six tau isoforms in the CNS; one of these (the inclusion of the optional microtubule-binding repeat coded by exon 10) is already highly relevant for tau pathology (Spillantini and Goedert, 2013). The picture is complicated by the ∼80 possible phosphorylation sites on tau, and the lively interplay of kinases and phosphatases that alter tau’s phosphorylation pattern (Grundke-Iqbal et al., 1986; Iqbal et al., 2016). A vast number of other possible post-translational modifications, such as truncation (Novak et al., 1993), nitration (Horiguchi et al., 2003), glycation (Ledesma et al., 1994), glycosylation (Wang et al., 1996), and ubiquitination (Mori et al., 1987) further expands the diversity of tau. Furthermore, the function of tau differs based on the molecule’s location – axonal (Méphon-Gaspard et al., 2016), dendritic (Ittner and Ittner, 2018), nuclear (Paholikova et al., 2014), or extraneuronal (Yamada et al., 2014; Sato et al., 2018). Finally, as an intrinsically disordered protein, tau behaves in a fundamentally different fashion when bound to interaction partners in contrast to being unbound in solution (Avila et al., 2016). These modifications create a pool of diverse tau molecules – the tau proteome. Different tau proteomes exists in health and disease. Thus, while “AD tau proteome” denotes the entire range of tau forms and derivatives present in Alzheimer’s, the term “physiological tau proteome” denotes solely the range of tau forms present in a brain in the absence of tauopathy. The overlap of these proteomes is substantial. It is likely that the entirety of the healthy proteome continues to exist in disease (albeit changed in quantity), along with a vastly expanded diseased proteome. Still, the idea that certain tau moieties are lost in disease may be worth investigating.

The burden of post-translational modifications of tau protein causes the transition of tau from its highly soluble physiological form into insoluble aggregated pathological forms. Hyperphosphorylation (Arif et al., 2014), truncation (Zilka et al., 2012b), or a combination of both are likely responsible, changing the disorder of tau into a mis-disordered pre-aggregation state, which then leads to fibril formation (Kovacech et al., 2010). The initiating factor behind these changes is unknown as of now, though it is likely that multiple endogenous and environmental factors lead to the formation of pathological tau naturally. Genetic (Lambert et al., 2013) and lifestyle factors (Baumgart et al., 2015) will determine the neurons’ proteostatic capacity and general resilience against tauopathy, and the size of the burden. For example, psychological stress and stress-induced kinases were shown to increase the phosphorylation burden of tau (Rissman et al., 2012; Lopes et al., 2016). Once the combined burden exceeds the proteostatic capacity (Balch et al., 2008) of vulnerable neurons, the patient’s brain begins its descent into tauopathy. Miniscule differences in the production or degradation of pathological tau can have a massive impact on its quantity (Gsponer et al., 2008), upsetting the status quo. Tau pathology results in a loss of physiological function and gain of toxic function. While in health, tau is vital to microtubule assembly (Weingarten et al., 1975), cellular transport (Klein et al., 2002), and DNA protection, diseased tau instead creates oligomers and fibrils (Wischik et al., 1988), destabilizes microtubules (Zilka et al., 2006), damages the proteasome (Opattova et al., 2013), causes neuronal and synaptic loss (Gómez-Isla et al., 1997; Tai et al., 2012) and promotes neuroinflammation (Zilka et al., 2012a). Seeding-capable tau molecules (“tauons”) convert healthy tau into pathological forms, and propagate tau pathology from cell to cell via multiple pathways (Novak et al., 2011; Mudher et al., 2017). Tau pathology results in axonal destabilization and impairment of transport (Wang and Mandelkow, 2016). Cytoskeletal changes, rarefication of neuronal processes, axonal retraction, and finally neuronal death follow (Braak et al., 1994; Kanaan et al., 2013).

The diversity in pathological tau forms entails a similar diversity in tau lesion distribution, morphology, and composition (Sergeant et al., 2005; Fitzpatrick et al., 2017; Falcon et al., 2018) (see Table 1). The phenotype of AD and non-AD tauopathies aligns with the distribution of neurofibrillary lesions, and the severity of impairment is tied to the amount of tau pathology present (Dickson et al., 2011; Josephs et al., 2011; Nelson et al., 2012). Specifically in AD, the lesion spreading pattern inversely recapitulates the myelogenesis of the brain (Braak et al., 2006), hinting at a selective vulnerability of flexible, poorly myelinated neurons with limited perineuronal support nets (Morawski et al., 2010) to AD pathology. Patterns of vulnerability to other types of tau pathology likely influence the lesion distribution in other tauopathies. For example, brain regions predominantly affected by the 4R-tauopathy PSP express a higher ratio of 4R-tau even in healthy controls (Majounie et al., 2013).

**Table 1:** Overview of the most prevalent tauopathies.

Disease	Tau lesion type	Lesion distribution	Phenotype	Reference
Alzheimer’s disease	Neurofibrillary tangles Neuritic component of plaques Neuropil threads 3R+4R tau	Allocortex, limbic regions, most neocortical fields except motor cortex, brainstem (locus coeruleus, raphe nuclei)	Predominantly amnestic, w. disorientation in time and space. Language, dysexecutive, and visual variant exist. Multiple domains impaired in dementia.	Pathology: (Braak and Braak, 1991) Clinics: (Morris et al., 2014; Jack et al., 2018)

Argyrophilic grain disease	Argyrophilic grains, pre-neurofibrillary tangles in neurons and coiled bodies in oligodendrocytes 4R tau	Mainly entorhinal cortex and hippocampus, insula, amygdala, hypothalamus, cingulated gyrus, …	Amnestic and multi-domain cognitive impairment. Behavioral abnormalities and FTD-like symptoms possible.	Pathology: (Grinberg and Heinsen, 2009) Clinics:(Rodriguez et al., 2016)

Chronic traumatic encephalopathy	Neurofibrillary tangles and neurites, astrocytic tangles 3R+4R tau	Focal (perivascular) epicenters, amygdala, hippocampus, nucleus basalis of Meynert, substantia nigra, locus coeruleus	Mainly executive function, behavior, and short-term memory impaired	Pathology: (McKee et al., 2013) Clinics: (McKee et al., 2013)

Corticobasal degeneration	Pre-tangles Astrocytic plaques thread-like lesions, corticobasal bodies, and coiled bodies 4R tau	Basal ganglia, thalamus, focal parietal, mostly asymmetric, and brainstem	Asymmetric levodopa-resistant parkinsonism, postural instability, dystonia, myoclonus. Behavioral changes, aphasia, cognitive impairment. Cortical sensory loss. Alien limb.	Pathology: (Josephs et al., 2011) Clinics: (Armstrong et al., 2013; Levin et al., 2016)

Dementia in Down syndrome	Similar to AD	Similar to AD	Similar to AD, complicated by existing cognitive disability	Pathology: (Hof et al., 1995) Clinics: (Ghezzo et al., 2014)

Frontotemporal lobar degeneration (w./w.o. Parkinsonism)	(extremely varied) 3R, 4R, or 3R+4R tau	Frontal and temporal/temporoparietal neocortex, (substantia nigra)	Language (PPA), behavior, executive function (bvFTD), possibly parkinsonism (FTDP-17)	Pathology: (Seilhean et al., 2011) Clinics: (Wszolek et al., 2006; Gorno-Tempini et al., 2011)

Pick’s disease	Pick bodies Astroglial processes 3R tau	Frontal and temporal neocortex, dentate gyrus, deep gray matter and brainstem (monoaminergic nuclei)	Language, behavior, executive function (as bvFTD or PPA)	Pathology: (Sieben et al., 2012) Clinics: (Hodges et al., 2004; Warren et al., 2013)

Progressive supranuclear palsy	Globose NFTs Tufted astrocytes 4R tau	Predominantly in basal ganglia, subthalamic nucleus, and substantia nigra. Involvement of motor cortex and cerebellum (e.g., superior cerebellar peduncle).	Vertical supranuclear gaze palsy, postural instability, falls, pseudobulbar palsy, rigidity, akinesia; behavior, executive function, and language impaired; Parkinsonism	Pathology: (Josephs et al., 2011) Clinics: (Levin et al., 2016; Respondek and Hoglinger, 2016)

Tangle-only dementia	Neurofibrillary tangles Neuropil threads Ghost tangles 3R+4R tau 4R→3R shift during tangle maturation	Allocortex and medial temporal lobe, limbic regions; subcortical: locus coeruleus, nucleus basalis of Meynert, amygdala	Mainly memory deficit; slower progression than AD	Pathology and Clinics: (Jellinger and Bancher, 1998; Jellinger and Attems, 2007)


*Most common lesion distribution patterns listed. Coexistence of tau pathology with multiple other pathologies is possible. The listed phenotype mentions possible clinical manifestations; not all signs are required for diagnosis. Lesser amounts of 3R-tau can be found in 4R-tau dominated pathologies, and vice versa*.

Numerous less well known and less common disorders with tau lesions other than those mentioned in Table 1 exist, e.g., familial British dementia and familial Danish dementia (Vidal et al., 1999), Parkinsonism-dementia complex of Guam (Arif et al., 2014), and many others (Spillantini and Goedert, 2013).

To counteract tau pathology via immunotherapy, it is necessary to characterize the pathological tau proteome in the target disorder(s), find the proper epitope(s) that would allow the capture of pathological tau moieties *en masse* and in their entirety, and interfere in the propagation of tau pathology. The mechanisms of action being discussed by the field include inhibition of aggregation and dis-aggregation, opsonisation and phagocytosis, immobilization of tau seeds and uptake inhibition, binding-mediated conformational change, tau re-distribution, and overall tau reduction.

## Epitope Selection

Due to the flexibility of the tau molecule, and due to the plethora of post-translational modifications that occur in health and in disease, the epitope landscape of tau is exceedingly rich. Not every epitope appearing on pathological tau is a viable immunotherapy target, though. Obviously, epitopes that predominantly appear on pathological tau, or are better accessible in pathological tau molecules than in their healthy counterparts should be preferred, as opposed to indiscriminately removing any tau. Reducing healthy tau protein was found to have detrimental effects (Marciniak et al., 2017), and (presumably) diluting the effect of the antibody between healthy (irrelevant) and pathological tau forms could reduce the overall efficacy of the treatment.

It is well known that truncation of tau’s N- and C-terminus promotes the molecule’s transition from its healthy state into pathological forms (Zilka et al., 2012b). Tau aggregation occurs inevitably via the microtubule binding region (MTBR) (Fitzpatrick et al., 2017), and in line with this mechanism, the only part of tau that is retained in all tau molecules that constitute and propagate neurofibrillary pathology is the MTBR. Conspicuously, tau fragments in the CSF of AD patients meanwhile rarely if ever contain the MTBR (Sato et al., 2018). Under physiological conditions, tau in the extracellular space in the brain is either full-length or truncated at the C-terminus. It is highly possible that tau containing MTBR is rapidly degraded in order to prevent formation of tau oligomers. Phosphorylation can speed up the process of tau degradation suggesting its physiological role in tau turnover (Sato et al., 2018). On the other hand, one or both termini of tau are absent in an important subset of pathological tau forms in the AD brain (Zilka et al., 2012b; Zhou et al., 2018), which thus escape immunotherapies aimed at epitopes on termini. The fact that tau becomes progressively more truncated (e.g., first at Asp421, then at Glu391) (Binder et al., 2005) illustrates that there may be a similar limitation to immunotherapies aimed at these *new* termini.

Other post-translational modifications of tau protein are similarly potential targets – whether phosphorylation, ubiquitination, glycation, glycosylation, or nitration, they all create novel epitopes on tau. The main limitation here is that many of these modifications are subject to a large degree of fluctuation. The main challenge is characterizing their distribution across the pool of all pathological tau moieties over the course of the disorder.

Conformation is an often-overlooked aspect of tau pathology (Novak et al., 2018b). Some epitopes may arise purely due to the alteration of the molecule’s conformation, and accessibility of individual epitopes may differ based on the binding partners, current conformation, and aggregation status of tau.

Location of the targeted tau moieties is also a crucial aspect. It is evident that intracellular and extracellular tau, and the tau molecules that pass into the CSF differ. In comparison to immunotherapies targeting extracellular pathology, such as Aβ, tau-targeted immunotherapy faces an additional challenge: many types of tau are located intracellularly [whether axonal, dendritic (Ittner and Ittner, 2018), somatic, or nuclear (Paholikova et al., 2014)]. The opinions differ on whether the intracellular compartments are accessible to immunotherapy, though. Tau responsible for intercellular spread has to pass through extracellular space, where it is accessible to immunotherapy (Evans et al., 2018) – unless it utilizes solely tunneling nanotubes. Also, we suspect that neurons outsource a portion of tau to their supporting cells for processing (Morawski et al., 2010; Mohamed et al., 2014); this tau could also be targeted in the extracellular space.

Finally, there is a subset of epitopes that are truly unsuited as immunotherapy targets. Antibodies binding to these epitopes were shown to promote tau aggregation *in vitro* (Kontsekova et al., 2014b). The promotion of tau aggregation occurred also in cell-free solution; thus, the most likely explanation is that antibodies against certain tau epitopes serve to stabilize a pathological tau conformation that promotes further aggregation.

Summarily, the presence of an epitope on pathological tau is not sufficient to make it a good immunotherapy target. It is necessary to know what the function of individual (pathological) epitopes is, what targeting a certain epitope means for the tau molecule, and what downstream mechanisms are engaged after antibody binding.

A range of assays can serve to evaluate the properties of candidate antibodies. It is not sufficient to demonstrate the antibody’s affinity to tau constructs or extracts in ELISA or similar methods. Ideally, for comparability purposes, it should be shown which parts of the tau proteome the antibody targets (e.g., both low molecular weight fragments, and higher molecular weight oligomers and fibrils). A range of seeding and aggregation assays can inform about the impact of immunotherapy on tau aggregation and fibrillisation. If an antibody is intended to opsonise tau for removal by the immune system, then its performance in phagocytosis promotion assays (e.g., assessed by flow cytometry) should be adequate. Microtubule assembly assays can be used to evaluate whether the antibody inhibits the cytoskeleton-destabilizing properties of pathological tau. Various seeding and tau spreading assays can be utilized to evaluate the antibody’s capability to halt the spread of tauons (Nobuhara et al., 2017).

## Characterization of Therapeutics and Models

In recent years, the number of publications on the role of tau protein in neurodegenerative disease, its (patho)physiology, and its role in diagnosis has skyrocketed – but often, the comparability of studies remains low. For the tau field to move forward in a consistent fashion, certain standards of methodology need to be established.

Comparability of immune response between studies (preclinical, clinical) is very low due to poor reporting standards for immunological outcomes (Agadjanyan et al., 2015). For active immunotherapies, the reporting of titre should be standard – using arbitrary ELISA units obscures the true antibody response, and makes comparisons all but impossible. Absolute quantification of antibody amount per volume would be ideal, as methodological issues (e.g., chromogen, secondary antibody) can confound titre measurements. Furthermore, for antibodies elicited in patients by the immunotherapy, the reactivity with various substrates needs to be evaluated thoroughly. Similarly, for passive vaccines, what the antibody targets and what it does not should be made perfectly clear (Table 2).

**Table 2:** Proposed substrates for reactivity assessments of tau-targeted immunotherapies.

Substrate	Description and details
The immunogen	Reference substrate

Healthy tau protein	Physiological tau, ideally both 3R and 4R (e.g., 2N3R, 2N4R)

Post-translationally modified tau	Truncated tau species, phospho-species

Sarcosyl-insoluble tau	Qualitative assessment in Western blot, quantitative assessment in ELISA, comparison to pan-tau antibody

Human brain tissue	Tissue immunohistochemistry on AD and non-AD tauopathies


For all tau-targeted therapies, what should be established is a comparability of models. Authors generally speak of models ‘developing tau pathology’ and the tau pathology being treated by their compound of choice. The average AT8-positive clump in a cell tells little about the nature of tau pathology in a given model, and its relevance to human disease. Models currently employed by the field are exceedingly diverse, differing in transgene (almost exclusively various forms of mutated tau), genetic background (multiple crossings of various mouse lines generating extensive variability of the phenotype), and expression level (related to the promotor), and thus it is not surprising that the resulting pathology differs as well (Skrabana et al., 2017). For example, numerous models don’t develop sarcosyl-insoluble tau, or higher order aggregates; argyrophilia or thioflavin-S reactivity is frequently absent. In other models, the transgene lacks the ability to sequester endogenous rodent tau. Models based on seeding tau pathology with exogenous material sometimes use recombinant tau fibrils, but human tauon strains are best replicated by utilizing genuine brain homogenates from human disease (Guo et al., 2016). While sequence homology between human and rodent tau is high, there are important differences in sequence and function, which may impact whether particular disease mechanisms can be modeled in rodents at all (Hernandez et al., 2018). The questions are obvious. How predictive are models with little tau aggregation for antibodies that aim to stop tau aggregation in human disease? How predictive are models that lack β-sheet structure in tau for therapies that aim to target human tau pathology that is arranged in β-sheets (Fitzpatrick et al., 2017)? Do we sufficiently account for the fact that a large portion of animal models of tau pathology is based on mutant tau, whereas most human tauopathy cases and all AD cases are not caused by a tau mutation? Finally, few authors investigate the impact that genetic background of the transgenic animals can have on the effects of a transgene; these effects can be pronounced (Stozicka et al., 2010).

To evaluate the properties of a model, and judge its suitability for preclinical efficacy assessment of tau-targeted immunotherapies, at least the features in Table 3 should be reported.

**Table 3:** Proposed aspects of model characterization.

	Model attribute	Method	Reference
Biochemical aspects	Tau proteome	Western blot • Sarcosyl-insoluble extracts • Antibodies specific for post-translational modifications • Antibodies specific for endogenous and transgenic/injected tau	Zilka et al., 2006; Yu et al., 2009; Forest et al., 2013
	
	Filament morphology	Electron microscopy	Crowther and Goedert, 2000;Ksiezak-Reding and Wall, 2005; von Bergen et al., 2006
	
	Pathology distribution and morphology	Immunohistochemistry • Antibodies specific for post-translational modifications • Antibodies specific for endogenous and transgenic/injected tau	Braak and Braak, 1991; Zilka et al., 2006; Josephs et al., 2011
	
	Presence of β-sheets	Staining with Thioflavin-S, Congo-red, silver	Vallet et al., 1992
	
	Seeding capability	*In vitro* (solution, cell assays) and *in vivo* (stereotaxic) seeding	Audouard et al., 2016; Courade et al., 2018
	
	Transgene expression level	Quantitative real time PCR (mRNA), Western blot, ELISA (protein), both related to the expression of endogenous tau	Koson et al., 2008; Filipcik et al., 2012

Clinical aspects	Impairment phenotype	Cognitive and motor assays, EEG, electrophysiology	Webster et al., 2014; Menkes-Caspi et al., 2015; Wolf et al., 2016
	
	Age of onset, survival	–	Stel et al., 2011
	
Other	Variability	Numerical variability in all of the above aspects must be quantified, with special attention to the effects of sex and genetic background.	–
	
	Neuronal loss	Quantification of neuron specific markers	West et al., 1991
	
	Synaptic loss	Quantification of synaptic markers	Robinson et al., 2014
	
	Neuroinflammation	Activation of astrocytes and microglia, cytokine profile	Stozicka et al., 2010; Kovac et al., 2011; Zilka et al., 2012a; Heneka et al., 2015;


Beside the attributes outlined in Table 3, certain additional aspects should be reported about specific types of models. For models designed utilizing viral vectors (e.g., Bolognin et al., 2012), an eventual impact of the vector itself should be analyzed and reported. The type, percentage, and distribution of transfected cells, as well as persistence of transfection should be evaluated. For models based on the administration of seeding-capable tau protein (e.g., Clavaguera et al., 2009; Guo et al., 2016), the seed should be thoroughly biochemically characterized, and the resulting tau pathology compared to it. Any methods used to ‘pre-condition’ the recipient animal (such as a transgenic background expression of human tau) should be reported; ideally, the impact of said pre-conditioning should be evaluated by comparing the results to the same procedure in wild-type animals. For models that develop pathology due to their transgene alone, the additional impact of administration of seeding-capable tau can be difficult to evaluate. On the contrary, when using models where expression of diseased tau is widespread, the researcher will have to address whether and how spreading mechanisms can be evaluated in this type of animal.

## Immunotherapies in Clinical Development

### Epitope Selection

Close to a dozen tau-targeted immunotherapies have been advanced to clinical trials since the debut of AADvac1 in 2013 (Kontsekova et al., 2014a). Differences in design philosophy of these immunotherapies are evident.

The active AADvac1 vaccine induces specific antibodies targeting 3 or 4 conformational epitopes on mis-disordered tau protein with an exposed microtubule-binding repeat domain, such as appear on truncated, oligomeric, and aggregated 3R/4R tau. The antibodies raised by the vaccine preferentially target the pathological forms of tau (Kontsekova et al., 2014a,b, Novak et al., 2017). Targeting of the four epitopes located throughout the microtubule-binding region is advantageous, as all aggregation of tau occurs via this domain (Fitzpatrick et al., 2017), and by targeting it, antibodies elicited by the vaccine are likely to both inhibit further aggregation, and capture all aggregation-capable tau species.

The recently unveiled monoclonal antibody UCB0107 also focuses on the central region of tau protein (residues 235–246 in close proximity of the microtubule-binding region), with a similar primary goal of inhibiting seeding-capable tauons, and thus preventing the spread of neurofibrillary pathology^1^.

Similarly, the monoclonal antibody LY3303560 derived from the MC-1 antibody (Jicha et al., 1997), reactive with aa313-322 of the third repeat domain, but also dependent on the aa 7–9 of tau’s N-terminus for binding, is being clinically developed. The antibody’s more extensive binding site in the repeat region is close to the ^306^VQIVYK^311^ β-sheet forming motif (von Bergen et al., 2006) which is flanked from the other side by the AADvac1-targeted epitope ^294^KDNIKHVPGGGS^305^. Therefore, the mechanisms of action of LY3303560 and AADvac1-induced antibodies may have commonalities, e.g., affecting the aggregation of tau, and capturing tauons which inevitably have this domain, though LY3303560 will miss any N-terminally truncated tau species (Courade et al., 2018).

The C-terminus of the molecule, particularly the serine residues S396/S404 that become differentially phosphorylated especially in pathological tau, were chosen as the target for the active vaccine ACI-35 (Theunis et al., 2013). In their later work, the authors reveal that the induced antibody response mainly recognizes the pS396 epitope, along with conformational change as factors distinguishing between healthy and pathological tau protein (Theunis et al., 2017). It is uncertain whether antibodies recognizing pS396 alone target the same (or similar) pool of tau as recognized by the PHF1 antibody, though.

Both AADvac1 and ACI-35 focus strongly on the selectivity of the antibodies for pathological tau. In combination with the over-abundance of pathological tau over physiological tau in the AD/tauopathy brain, and considering the fact that pathological tau will be over-represented in the extracellular space, off-target reactivity against healthy tau will be greatly reduced. Not all tau-targeted immunotherapies focus as much on this property, with some being content to target any extracellular tau.

The monoclonal IgG1 antibody BIIB076 (Czerkovicz et al., 2018) is classified as a pan-tau antibody and is reported to target monomeric and fibrillary forms of tau pathology, without regard to whether they are specific for AD.

ABBV 8E12 targets aa25-30 at the N-terminus of tau with the aim to clear extracellular forms, and thus inhibit seeding (West et al., 2017). Another monoclonal antibody targeted at the N-terminus, the IgG4 antibody BIIB092, focuses on N-terminal fragments of tau (which likely arise during tau truncation) with the aim of reducing their secondary impact on amyloid-β processing and pathology, and countering neuronal hyperactivity (Bright et al., 2015); the antibodies are thus bound to miss any N-terminally truncated tauons, though. Similarly, the RO 7105705 antibody aims at the N-terminus of extracellular tau, and does not place importance on discrimination between tau forms specific to AD/tauopathy and those observed in healthy brains.

The jury is still out on whether tau-targeted antibodies actually are able to enter neurons or not. One group reports this is the case (Gu et al., 2013), while numerous others don’t report any passage of antibodies into neurons. In our in-house studies, no passage of tau-targeted antibodies into intact neurons was observed as of today. The final answer to this question is especially relevant for tau-targeted immunotherapies that don’t preferentially target diseased tau, as undesired interference with interneuronal transfer of non-pathogenic tau may have profound consequences (Bright et al., 2015; Evans et al., 2018).

Along with an understanding of the function of tau’s domains in health and disease comes the realization that it is likely not sufficient to target any epitope on tau to have an impact on the progression of neurofibrillary pathology. The abovementioned immunotherapies will target different pools of tau, with different effects on the molecule upon antibody binding. Similarly, due to their differing isotypes, the downstream effects of antibody binding to tau will also differ.

### Isotype Selection

Human IgG isotypes differ pronouncedly in their functions, especially in the downstream effects they initiate after binding, but also in their avidity and propensity to generate immune complexes. Especially some IgG subclasses (IgG1, IgG3) are adept at engaging downstream innate mechanisms, such as complement and phagocytosis by microglia (Vidarsson et al., 2014). Based on very limited evidence (Lee et al., 2016), a portion of the AD immunotherapy field seems to be laboring under the impression that engaging the innate immune system is inherently and invariably dangerous, and thus gravitates toward using IgG4 and modified backbones with reduced effector functions for their antibodies (Table 4). Extrapolating from *in vitro* isolated microglial culture from young rodents to elderly humans whose microglia are embedded in a complex network of interaction with other cells seems is unjustifiable though. They are fundamentally different entities (Smith and Dragunow, 2014); microglia that produce neurotoxic inflammatory mediators in culture may behave in a different manner in the living brain. It is indeed likely that microglial senescence and the ensuing reduced capacity of microglia to support neurons and phagocyte tauons are permissive factors involved in the pathogenesis of AD (Streit et al., 2009). Microglia probably are victims of the neurodegenerative process who actually need aid (e.g., in the form of tau-targeting antibodies) to properly oppose the progression of AD. We believe that the microglia can be an extremely valuable ally in combating neurofibrillary pathology, if they are engaged properly (Novak et al., 2018b). The benign safety profile observed in studies of AADvac1 (Novak et al., 2017) (which induces an IgG1-dominated antibody response) indicates that pathological tau can be targeted safely with IgG1 antibodies.

**Table 4:** Overview of tau-targeted immunotherapies in clinical development.

Compound	Type and isotype	Epitope	Targeted tau species	Proposed mechanism	Disease models used
AADvac1	Active, antibody response is mostly IgG1	Primary: 294–305 Secondary: 268–283; 330-335; 362–367	Conformationally altered extracellular tau (monomer, oligomer, fibril), focusing on seeding-capable extracellular tau	Anti-aggregation, opsonisation, inhibition of cell-to-cell spread of neurofibrillary pathology *Ref.:* (Kontsekova et al., 2014a,Kontsekova et al., 2014b; Novak et al., 2017)	SHR72 rats (tau 151-391/4R), SHR background R3/m4 mice (tau 151-391/34R), C57BL/6 background

ACI-35	Active	pS396 (some sources additionally mention pS404)	Tau pS396 with a conformation that is typically present in the brain of tauopathy patients, preferentially multimeric conformers	Reduction of pS396-tau (soluble and insoluble) *Ref.:* (Theunis et al., 2013)	P301L tau mice, FVB/Nbackground

ABBV-8E12	Passive, IgG4	25–30	Extracellular tau	Inhibition of cell-to-cell spread of neurofibrillary pathology *Ref.:* (Yanamandra et al., 2015)	P301S tau mice, B6C3 background

BIIB076	Passive, IgG1	Undisclosed	Monomeric and fibrillary forms, as well as tau isolated from healthy human and Alzheimer’s disease brains.	(undisclosed) *Ref: alzforum.org*	(Undisclosed)

BIIB092	Passive, IgG4	Exact epitope undisclosed (N-terminal inserts?)	extracellular, N-terminal fragments of tau (eTau)	Secondary reduction of amyloid-β and of neuronal hyperactivity *Ref: alzforum.org*	JNPL3 P301L mice, C57BL/DBA2/SW background tau-4R/2N-P301L van Leuven mice, C57Bl/6 background

JNJ-63733657	Passive, isotype undisclosed	Undisclosed, presumably mid-region	Seeding-capable tau	Neutralization of tau seeds and reduction of pathology *Ref: alzforum.org*	(Undisclosed)

LY3303560	Passive, isotype undisclosed	7–9; 313–322	Soluble tau aggregates with intact N-terminus	Neutralization of tau seeds and reduction of pathology. *Ref: alzforum.org*	(Undisclosed)

RO 7105705	Passive, IgG4	N-terminus	N-terminus of all six isoforms, both monomeric and oligomeric, regardless of phosphorylation status	Inhibition of cell-to-cell spread of neurofibrillary pathology *Ref: alzforum.org*	(Undisclosed)

UCB0107	Passive, isotype undisclosed	235–246	Seeding-capable extracellular tau	Inhibition of cell-to-cell spread of neurofibrillary pathology *Ref:* (Courade et al., 2018), *alzforum.org*	(Undisclosed)


Epitopes numbered according to the 2N4R tau isoform.

It is essential to clarify whether it is sufficient to bind the targeted tau moiety via a monoclonal or vaccine-induced antibody, or whether downstream mechanisms such as Fc-receptor mediated phagocytosis are necessary for (or greatly contribute to) efficacy. While all monoclonal antibodies currently in clinical development are of the IgG1 or IgG4 isotypes, a case is to be made also for IgG3, whose greater flexibility of the Fab arms may promote avidity and antigen-binding capacity (Vidarsson et al., 2014). As a trade-off, this isotype promotes complement activation the most; should this trait be undesirable, there are ways to mitigate it (Saxena and Wu, 2016). As for IgG4, one fact which must be kept in mind is that monovalent bispecific antibodies will result from the IgG4 Fab arm exchange (Schuurman et al., 1999), consisting of one light and one heavy chain of a random endogenous IgG4 antibody, and one half of the administered IgG4 monoclonal. These antibodies are unable to crosslink the same antigen – a property which may become important in the inhibition of tauons.

### Mechanisms of Action

Generally, reports of tau-targeted immunotherapies focus on evaluating clinical and biochemical endpoints in animals and humans (test performance, biomarkers, atrophy, tau load and neurofibrillary tangle counts), and assessments of mechanism of action are relegated to *in vitro* assays.

The current understanding of the propagation of tau pathology highlights possible avenues by which an immunotherapy could intervene. At first, pre-aggregate forms of pathological tau are generated intracellularly. To target this step, antibodies would have to be able to enter neurons – this may or may not be possible. Once intracellular pathology is sufficiently advanced, the affected neuron begins to shed tau seeds – the tauons (Mudher et al., 2017). Being (likely) extracellular (Novak et al., 2011), they are well-accessible to immunotherapy. Antibodies may be able to simply immobilize them by binding; disassembly of existing early-stage aggregates is also conceivable if the antibody is a more preferable binding partner for pathological tau than other tau molecules (Kontsekova et al., 2014b). The pathological conformation of tau molecules may be changed as antibodies bind them (another form of template-mediated conformational change, with the antibody acting as template/chaperone) (Sarkar et al., 2008). Via their effector functions, antibodies are able to engage immune cells, such as microglia, to phagocyte and degrade pathological tau (Bournazos et al., 2015). Antibodies may protect cells by preventing the binding of tauons to receptors that are needed for internalization (Evans et al., 2018). Finally (again, only if antibodies are able to enter neurons), template-mediated aggregation induced by tauons entering healthy neurons could be prevented via an antibody obstructing the aggregation process (Kontsekova et al., 2014b).

We have recently reported the proposed mechanism of AADvac1 (Novak et al., 2018b). In line with the above considerations, the goal of AADvac1 treatment is to induce antibodies that block the spread of tauopathy by immobilizing tauons and opsonising them for removal by the immune system, e.g., microglia. Mechanisms of other tau-targeted immunotherapies in clinical development are detailed in Table 4.

### Results of Animal Studies

Where reported, the results of preclinical animal studies of the immunotherapies in Table 4 showed that the animal model of choice was treated with the compound, whereupon its tau pathology was reduced, and clinical symptoms improved. The significance of these findings is mostly determined by the validity of the treated animal model. The details of these results warrant further analysis, though.

Treatment of SHR72 transgenic rats with AADvac1 resulted in ∼70% less insoluble tau accumulation in their brains; interestingly, the amount of pathologically hyperphosphorylated tau was reduced by 90-98% (Kontsekova et al., 2014a). While the conformational epitope targeted by AADvac1 is phosphorylation-independent, the pronounced reduction in pathological phospho-tau species may indicate that conformationally altered tau is more liable to be hyperphosphorylated. The antibody response in the treated animals preferentially targeted pathological tau 151-391/4R, in comparison to physiological tau 2N4R, and was able to recognize tau pathology in all evaluated Alzheimer brains (Kontsekova et al., 2014a). Both of the above were replicated in humans (Novak et al., 2017, 2018a). The administration route was identical in animals and humans (subcutaneous injection). Transgenic animals received five doses of 100 μg peptide-KLH conjugate, with ∼16 μg tau peptide per dose. Humans received a higher dose in the first-in-man study (40 μg peptide coupled to KLH). Treatment alleviated approximately 50% of the animals’ impairment, as measured by the Neuroscale test battery (Korenova et al., 2009; Kontsekova et al., 2014a).

Animal studies of ACI-35 included the evaluation of safety, immunogenicity, and efficacy in Tau.P301L mice. Following subcutaneous administration, the elicited antibodies recognized tau pathology in aged GSK3bxTauP301L mice; whether the antibodies are able to bind human tau pathology was not evaluated in the initial study (Theunis et al., 2013). The authors’ later work indicates that the monoclonal antibody generated using ACI-35 recognizes human NFTs (Theunis et al., 2017). The evaluated safety parameters, e.g., T-cell response, gliosis, and various other inflammatory markers did not indicate any undesired activity of the immune system. Animals received a total of four doses; the exact tau peptide dose is not discernible from the manuscripts (Theunis et al., 2013). The treatment resulted in a ∼25% reduction of soluble pS396 phospho-tau, and a ∼33% reduction of sarcosyl-insoluble pS396 phospho-tau. Curiously, even though the immunogen also featured the pS404 phospho-epitope, the pS404 phospho-tau levels were not markedly affected by ACI-35 treatment; neither was insoluble pS396 phospho-tau in the brainstem. Modest clinical improvement was seen on hind-limb clasping, body weight, and survival (Theunis et al., 2013).

Weekly treatment of P301S mice with 50 mg/kg of the HJ8.5 antibody administered intraperitoneally over the course of 12 weeks resulted in pronounced reduction of formic acid soluble tau. Phospho-tau, as measured by the anti-pS202/pT205 antibody AT8, and thioflavin-S reactive pathology were affected as well. As P301S mice also suffer brain atrophy, the capacity of HJ8.5 to prevent this was assessed; the treatment resulted in a modest atrophy reduction. Improvements in sensorimotor function were also seen (Yanamandra et al., 2015). The humanized version of this antibody is in clinical trials under the name ABBV-8E12.

Differences in models and assessment methodology preclude direct efficacy comparisons of the above treatments, highlighting the need to establish assay and reporting standards. No publications on preclinical efficacy of the remaining compounds mentioned in Table 4 are available as of today.

### Results of Human Clinical Trials

All tau-targeted immunotherapies in clinical trials are at early stages of development. Only limited clinical trial data have been reported so far, and only for AADvac1 (Novak et al., 2017, 2018a).

Treatment with AADvac1 was able to elicit an IgG response against the tau peptide in 29/30 treated patients, and a response against truncated tau 151-391/4R in 25 of 28 patients where this response was assessed (Novak et al., 2017). More importantly, the elicited antibodies were able to stain sarcosyl-insoluble tau extracts from Alzheimer’s disease and other tauopathies, with all evaluated extracts being labeled by patient sera. Both low molecular weight fragments and high molecular weight aggregates were labeled. The same was seen in histological assessment, where patient sera were able to stain tau pathology in brains with Alzheimer’s disease, corticobasal degeneration, progressive supranuclear palsy, and Pick’s disease, highlighting the fact that the targeted conformational epitopes in the microtubule-binding region of tau represent a common denominator of tau pathology in a wide range of tauopathies (Novak et al., 2018a).

While the phase 1 trials were not designed as efficacy studies, inter-individual differences in AADvac1-induced antibody titres allowed the comparison between the strength of the antibody response and disease progression. A trend toward slower cognitive decline and lower hippocampal atrophy rate was observed in patients with higher titres (Novak et al., 2018a).

The safety profile in both studies was benign, with local injection site reactions being the only adverse event clearly tied to treatment (Novak et al., 2017, 2018a). The accruing results on AADvac1, and the fact that no drastic safety concerns are being reported for ACI-35 either indicate that active immunization can be a safe way to address tau pathology. The safety aspect will become increasingly important once the indication for active tau-targeted immunotherapies progresses from treating neurodegeneration toward preventing it.

## Immune Senescence

With active immunotherapies, an additional layer of complexity is added to development. The immunogen itself is not the molecule that is meant to mediate the therapeutic effect; rather, the antibodies induced by vaccination are the active agent. A vaccine depends on the patient’s immune system to deliver these antibodies, yet the vast majority of patients suffering from neurodegenerative disease are elderly, and their immune system senescent (Siegrist and Aspinall, 2009). Thus, a profound understanding of immunological mechanisms in the elderly is of paramount importance to ensure adequate antibody production, or to identify patients who can and who cannot benefit from a certain immunotherapy.

In the phase 1 study of AADvac1, we have investigated a range of lymphocyte populations, and evaluated their correlations with the antibody titres achieved at the end of the initial vaccination regimen (i.e., six doses in monthly intervals). The patient with the highest response had a ∼40× greater antibody titre than the patient who had the lowest IgG response. This diversity in response could, to an extent, be explained by differences in lymphocyte counts, T-helper cells counts, and the percentage off lymphocytes and neutrophil granulocytes (Novak et al., 2017). The ratio of lymphocytes to neutrophils could be interpreted as the immune system’s overall predisposition toward a T_H_1 or T_H_2 type of response, with the latter being more conductive toward an antibody-mediated response. The phase 1 dataset of 30 patients (with a sole non-responder) was too small to identify a cut-off for non-responders; it may be possible to establish one based on the ongoing phase 2 study (NCT02579252). Immunological cut-offs could be useful for the enrichment of trial populations for active immunotherapies. Immunological characterization of populations in clinical trials of immunotherapies could further promote the comparability of studies, and provide another important level of demographic information, analogous to age, sex, and ApoE genotype.

Another part of immune senescence applies equally to active and passive immunotherapies – that of the innate immune system (microglial senescence in particular) (Streit and Xue, 2014; Streit et al., 2014). Aged microglia undergo transformation from ramified to amoeboid morphology. They display a pro-inflammatory phenotype producing several inflammatory cytokines (Udeochu et al., 2016). An extensive genetic and proteomic study on aged microglia revealed that several pathways are deregulated during the aging of brain microglia including phagosome and endosome pathways, interferon signaling and antigen processing and presentation (Olah et al., 2018). If phagocytosis and antigen presentation are an inherent part of a vaccines or an antibody’s pharmacodynamics, then microglial senescence could introduce further variance into the response to therapy. Markers of microglial senescence in the CSF or periphery yet have to be identified, yet will be extremely useful in explaining variability in the treatment responses to tau-targeted immunotherapies.

## Vaccine Design

To avoid a deleterious T-cell response aimed at a self-antigen, as seen with the first anti-amyloid-β vaccine AN-1792 (Gilman et al., 2005), the viable approaches are either to outsource co-stimulatory T-cell epitopes to a carrier, as done for CAD-106 (Winblad et al., 2012) and AADvac1 (Kontsekova et al., 2014a, Novak et al., 2017), or to induce a T-cell independent antibody response (Theunis et al., 2013).

For T-cell dependent responses, the age-dependent reduction in T-cell repertoire (Britanova et al., 2014) is a challenge, yet can be overcome by adequate carrier selection. Carriers that present a great diversity of potential T-cell epitopes (e.g., KLH with its 400 kDa mass and minimal sequence similarity to humans) have a higher chance to be recognized at all. Another option is to use carriers which could benefit from existing immunological memory [e.g., tetanus toxoid (Pichichero, 2013), or artificial carriers that feature a broad spectrum of T-cell epitopes from various antigens (Davtyan et al., 2017)]. It is possible that early issues with the immunogenicity of the CAD106 vaccine were caused by the low epitope diversity of the bacteriophage Qβ carrier (Winblad et al., 2012).

Adjuvants are an essential component of active vaccines. For AADvac1,we have selected aluminum hydroxide (Novak et al., 2017) as aclassic T_H_2 response promoting adjuvant that performed well inpre-clinical studies, but the possibilities recently have expanded asthe field turns its attention to novel adjuvants. A limitation is that most of the novel adjuvantsfavor a T_H_1 response (Reed et al., 2013).

Ideally, each antigen should be tested with multiple carriers and adjuvants in humans to optimize the antibody response (Della Cioppa et al., 2015); as there in only so much we can learn from animal and *in vitro* studies about how the immune system of elderly humans operates, an empirical approach is warranted. On the other hand, full factorial study designs run into feasibility issues with the addition of further carriers and adjuvants as the number of study arms quickly grows unmanageable and statistical multiplicity increasingly obscures findings. A challenge for immunogenicity optimization in active immunotherapy is the need to assess immunogenicity in the target population, i.e., AD/tauopathy patients. It is known that with increasing age, immunological health plays an ever-increasing role in maintaining cognitive health; it is likely that Alzheimer patients will differ from cognitively healthy elderly also in their immune competence. Furthermore, the risk-benefit ratio for healthy elderly differs from dementia patients as the response to active vaccines cannot be easily terminated.

## Conclusion

The development of tau-targeted immunotherapies has recently entered an exciting period of rampant growth and innovation. At this early stage, much of the learning is empirical. To maximize the knowledge obtained from these early trials, certain methodological and reporting standards should be implemented, both for preclinical and clinical research. Animal models and patient populations should be well-characterized; so should the epitopes of immunotherapies and their mechanisms of action (putative and proven). The greatest benefit to the field would be a thorough characterization of the diseased tau proteome – what molecular properties are found in individual tau species, where these species are located (cellular compartment/extracellular), and what their effects are. This is certainly a daunting, Herculean feat, but of immense value for the understanding of the mechanism of tau-targeted therapies: “What portion of tau does an immunotherapy affect? How? What is the impact on the physiological and pathophysiological function of tau? What human data have been used to confirm this?”

The immunotherapies currently in clinical development appear to be based on a range of philosophies – from an overall reduction of tau, to inhibition of aggregation, and cell-mediated tauon clearance. The targeted epitopes similarly differ, with three broad groups (N-terminus, mid-domain and MTBR, and C-terminus) emerging. With minimal data, clinical or otherwise, being available at this stage of development, in-depth comparisons are not possible. Greater transparency in the field would promote the development of therapies, and shorten the time until we are able to offer an efficacious therapy to tauopathy patients.

## Author Contributions

All authors listed have made a substantial, direct and intellectual contribution to the work, and approved it for publication.

## Conflict of Interest Statement

PN, EK, NZ and MN are employees of AXON Neuroscience SE or its subsidiaries.

## Footnotes

1 www.alzforum.org
